# Natural course of behavioral addictions: a 5-year longitudinal study

**DOI:** 10.1186/s12888-015-0383-3

**Published:** 2015-01-22

**Authors:** Barna Konkolÿ Thege, Erica M Woodin, David C Hodgins, Robert J Williams

**Affiliations:** 1 Department of Psychology, University of Calgary, 2500 University Drive NW, Calgary, AB T2N 1N4 Canada; 2 Department of Psychology, University of Victoria, PO Box 3050 STN CSC, Victoria, BC V8W 3P5 Canada; 3 Faculty of Health Sciences, University of Lethbridge, 3017 Markin Hall, Lethbridge, AB T1K 3M4 Canada

**Keywords:** Behavioral addiction, Natural course, Spontaneous recovery, Prevalence, Prospective design, Sex differences, Help-seeking, Substance abuse comorbidity

## Abstract

**Background:**

Resolving the theoretical controversy on the labeling of an increasing number of excessive behaviors as behavioral addictions may also be facilitated by more empirical data on these behavioral problems. For instance, an essential issue to the classification of psychiatric disorders is information on their natural course. However, longitudinal research on the chronic vs. episodic nature of behavioral addictions is scarce. The aim of the present study, therefore, was to provide data on prevalence, substance use comorbidity, and five-year trajectories of six excessive behaviors—namely exercising, sexual behavior, shopping, online chatting, video gaming, and eating.

**Methods:**

Analyses were based on the data of the Quinte Longitudinal Study, where a cohort of 4,121 adults from Ontario, Canada was followed for 5 years (2006 to 2011). The response rate was 21.3%, while retention rate was 93.9%. To assess the occurrence of each problem behavior, a single self-diagnostic question asked people whether their over-involvement in the behavior had caused significant problems for them in the past 12 months. To assess the severity of each problem behavior reported, the Behavioral Addiction Measure was administered. A mixed design ANOVA was used to investigate symptom trajectories over time for each problem behavior and whether these symptom trajectories varied as a function of sex.

**Results:**

The large majority of people reported having problematic over-involvement for just one of these behaviors and just in a single time period. A main effect of time was found for each problem behavior, indicating a moderately strong decrease in symptom severity across time. The time x sex interaction was insignificant in each model indicating that the decreasing trend is similar for males and females. The data also showed that help seeking was very low in the case of excessive sexual behavior, shopping, online chatting, and video gaming but substantially more prevalent in the case of excessive eating and exercising.

**Conclusions:**

The present results indicate that self-identified excessive exercising, sexual behavior, shopping, online chatting, video gaming, and/or eating tend to be fairly transient for most people. This aspect of the results is inconsistent with conceptualizations of addictions as progressive in nature, unless treated.

**Electronic supplementary material:**

The online version of this article (doi:10.1186/s12888-015-0383-3) contains supplementary material, which is available to authorized users.

## Background

Addictions are chronic disorders with substantial adverse impact on both the individual and societal level. Further, the prevalence of addictions is among the highest of mental disorders, especially when behavioral addictions are also taken into account [1]. Although the range and criteria used by researchers and clinicians to describe behavioral addictions have been highly debated, there is an emerging consensus that non-chemical addictions are similar in their main characteristics to substance-related addictions [2]: they generate short-term rewards that promote behavioral persistence despite the knowledge of adverse consequences [3-5].

Employing these criteria, an increasing number of behaviors qualify as addictions ranging from the generally accepted (e.g., online gaming addiction [6,7]) through the more controversial (e.g., television and sex or pornography addiction [8-10]), to the highly speculative (e.g., love, tanning or shoplifting addiction [11-13]). Impairment of control over these behaviors leads to the neglect of interpersonal relationships, role obligations, and activities previously important; and often also results in negative health consequences ranging from minor sleep pathologies through obesity and functional somatic symptoms to even suicide [14-20]. The phenomenon of reduced control is one of the key elements, the presence of which makes it reasonable to conceptualize several excessive behaviors as behavioral (or process) addictions [5].

The increased openness to categorize certain excessive behaviors as addictions by researchers and clinicians of the field has received a cautious approval by mainstream psychiatry: the newest edition of the Diagnostic and Statistical Manual of Mental Disorders (DSM-5 [21]) already contains a Non-Substance-Related Disorders subcategory within the Substance-Related and Addictive Disorders class. At present, the subsection includes only one diagnosis, Gambling Disorder, which was relocated from the Impulse Control Disorders category of the DSM, where it was listed between 1980 and 2013 [22]. Disorders related to excessive eating were also considered to be reclassified from eating to addictive disorders in the DSM-5 [23], although ultimately they were retained in the eating disorders section. Further, Internet Gaming Disorder has been included in the Conditions for Further Study section in the fifth edition of the DSM, suggesting that this and most likely other behavioral addictions might receive official recognition in psychiatric nosological systems in the future.

On the other hand, the tendency to categorize an increasing number of behaviors as addictions, and thus medicalizing them, raises concerns: many professionals warn that as a result, the public will perceive psychiatric diagnoses as less serious in nature. Opponents of the expanded diagnostic system argue that ‘mental illness’ loses its meaning and relevance when a significant portion of the population falls into this category and when all atypical human behavior becomes medicalized [24]. Another perspective emphasizes that a high prevalence of *certain* types of addiction in the population (and an even much higher prevalence of *some* type of addiction) might suggest that addictions are a natural feature of human nature and therefore they should be interpreted much more as a lifestyle choice than psychopathology in itself [1]. Finally, there is concern that the medicalization of behavioral addictions drives our attention to the person instead of the societal, technological, and financial systems that may make individuals susceptible to the development of these dysfunctional habits [25].

Resolving the theoretical controversy on the labeling of an increasing number of excessive behaviors as addiction may also be facilitated by more empirical data on these behavioral problems. Lack of empirical data was an important factor behind the fact that only one behavioral addiction has been recognized in the DSM-5 and one other received a status explicitly calling for more empirical research (cf. [26]). For instance, an essential issue to the classification of psychiatric disorders and their management is information pertaining to their natural course and outcome. However, longitudinal research on the chronic vs. episodic nature of behavioral addictions is scarce, especially with follow-up times longer than 1 or 2 years [27-32].

The aim of the present study, therefore, was to provide data on the prevalence and natural course of six excessive behaviors – namely exercising, sexual behavior, shopping, online chatting, video gaming, and eating. The study also sought to explore potential sex differences in the course of behavioral addictions, which is an aspect often neglected in the literature, despite a substantial amount of accumulated data showing that men and women differ in terms of the occurrence, prevalence, correlates, and consequences of their addictive behaviors [33,34]. Finally, help-seeking was also investigated in relation to the six behaviors studied to better understand how individuals struggling with excessive behaviors try to cope with their difficulties and to compare these patterns with previous data on help seeking in substance addicts.

## Methods

### Sample and procedure

The analyses of the present paper are based on the data of the Quinte Longitudinal Study (QLS) [35], the study protocol of which was approved by the Human Subject Research Committee at the University of Lethbridge. The primary aim of the survey was to help the development of an etiological model of problem gambling; however, the diverse set of variables assessed allows the longitudinal investigation of other behavioral addictions as well. The authors of the present study obtained permission from all authors of the QLS to conduct the analyses presented in this paper. A cohort of 4,121 adults from Ontario, Canada was followed for a period of 5 years (2006 to 2011) in the QLS. Most important sociodemographic characteristics of the sample are summarized in Table 1.

**Table 1:** **Sociodemographic characteristics of the sample at baseline**

Sex		Employment status	
Female	2,254 (54.7)	Unemployed	195 (4.7)
Male	1,867 (45.3)	Retired	750 (18.2)
Age [M(SD) years]	46.1 (14.1)	Homemaker	227 (5.5)
Education		Full-time student	88 (2.1)
Some elementary	17 (0.4)	Sick or maternity leave	227 (5.5)
Completed elementary	31 (0.8)	Employed part-time	493 (12.0)
Some high school	414 (10.0)	Employed full-time	2,141 (52.0)
Completed high school	823 (20)	Annual household income	
Some postsecondary	899 (21.8)	<20,000 CAD	401 (9.7)
Completed technical school	207 (5.0)	20,000-29,999 CAD	490 (11.9)
Completed college/university	1,563 (37.9)	30,000-39,999 CAD	511 (12.4)
Professional degree/PhD	167 (4.1)	40,000-49,999 CAD	469 (11.4)
Marital status		50,000-59,999 CAD	436 (10.6)
Never married	491 (11.9)	60,000-69,999 CAD	392 (9.5)
Married	2,393 (58.1)	70,000-79,999 CAD	322 (7.8)
Common-law	551 (13.4)	80,000-89,999 CAD	282 (6.8)
Separated	207 (5.0)	90,000-99,999 CAD	209 (5.1)
Divorced	318 (7.7)	100,000-119,999 CAD	259 (6.3)
Widowed	161 (3.9)	120,000-149,999 CAD	152 (3.7)
		>150,000 CAD	101 (2.5)
		Missing	97 (2.4)

Recruitment was conducted via random digit telephone dialing from a pool of numbers with area codes and prefixes estimated to be within 70 kilometers of the city of Belleville. The response rate in the QLS was 21.3%, a value similar to those obtained in research with a similar focus e.g., [36]. However, retention rate in this study was 93.9%, an exceptionally high value in large scale longitudinal research of this nature. Two samples were recruited: a ‘general population’ sample (n = 3,065) and an ‘at risk’ sample for problem gambling (n = 1,056). For the person to be eligible to be invited to participate in the ‘at risk’ subsample, they had to indicate one or more of the following: (1) spending $10 or more per month on lottery, instant win tickets, bingo, casino table games, or games of skill against other people; (2) playing either slot machines or betting on horse racing in the past year; (3) an intention to gamble at a new slots-at-racetrack facility that was scheduled to be built sometime in the next few years in the area. The purpose of recruiting the ‘at risk’ subsample was to ensure that there were a sufficient number of people in the cohort who became problem gamblers during the course of the study. The detailed methodological description of the study can be found elsewhere [37].

If the person agreed to participate in the survey in the initial telephone call, he/she was sent an email with a link to the online questionnaire or booked into a time slot at the program office where they completed the survey on a computer on site. A total of 69.5% opted for their home computer in Survey 1, with this proportion steadily increasing to 90.0% in Survey 5. A small percentage of people completed a paper and pencil version of the survey because of their unfamiliarity and/or dislike of computers (1.2% to 1.9% depending on the survey year). The survey was re-administered to all cohort participants on an annual basis.

Because the focus of the Quinte Longitudinal Study was to examine changes in gambling over time, it was not essential that the sample be perfectly representative of the Ontario or Canadian population (only that it contained a diverse range of gamblers). Nonetheless, the demographic profile of the sample is fairly similar to Canadian adults (15+) as established by the 2006 Canadian Census, with the exception that the present sample tends to include slightly fewer people aged 18–24, seniors 65 and older, single people, and has a somewhat higher level of educational attainment (see Table 1). In addition, the attrition analyses concerning the target behaviors showed that those completing the test battery at all five data waves reported excessive exercising (χ^2^ = 7.11, p = 0.029, Cramer’s V = 0.042) and video gaming (χ^2^ = 13.56, p = 0.001, Cramer’s V = 0.057) less often than those missing at least one assessment occasion. With regard to excessive sexual behavior, shopping, online chatting, and eating, no difference was found between completers and non-completers.

### Measures

Only a subset of variables/instruments from the complex test battery of the Quinte Longitudinal Study were employed in the present analyses; these are described below. Sociodemographic characteristics analyzed were participants’ sex, age, educational level (with eight response categories), marital status (with six response categories), employment (with seven response categories), and household income (with twelve response categories).

Since the excessive behaviors assessed in the present study are not included in the current psychiatric nosological systems, there are no clear-cut diagnostic criteria and thus recognized gold standard measurement tools for their assessment. Previous research has shown however, that self-diagnosis is an appropriate and clinically meaningful way to assess the presence of addictive disorders [31,38,39]. Therefore, to assess the occurrence of behavioral addictions (other than gambling disorders) in the sample, a yes or no type question was used (‘Are there activities that you engage in where your over-involvement has caused significant problems for you in the past 12 months? Check off any that apply’). Six response options were offered at baseline: sex or pornography, exercise, shopping, Internet chat lines, video or Internet gaming, and other. Excessive eating was added as a category beginning in Survey 2, due to its frequent endorsement in the ‘other’ category in Survey 1.

To assess the severity of each problem behavior reported, the Behavioral Addiction Measure was administered to those participants who reported the presence of any excessive behaviors. This instrument is a 21-item scale adapted from the Problem and Pathological Gambling Measure [40,41]. The items of the instrument cover three domains: psychosocial problems caused by the behavior (13 items; e.g., interpersonal or financial difficulties), impaired control (3 items; spending more time or money on the activity than intended, intention to cut down or quit), and other addiction-related characteristics (5 items; e.g., craving, preoccupation). Higher scores indicate more severe addiction problems. The full text of the scale is available on the publisher’s website as a supplemental material for this article [see Additional file 1]. Internal consistency for the scale—measured by the Cronbach’s alpha coefficient—ranged from .77 to .92 depending on excessive behavior and time point.

Individuals were also asked about help seeking for each problematic behavior identified, using the following two questions: ‘Have you ever sought help for these problems? (yes/no)’ and ‘Where did you seek help from?’ (friends, family, family doctor, psychologist, psychiatrist, counseling service, pastor/minister/priest, and telephone help/hotline). Finally, substance use characteristics (presence versus absence of any substance abuse or dependence) were assessed using an adaptation of the Composite International Diagnostic Interview [42], the Alcohol, Smoking and Substance Involvement Screening Test [43], and the Problem and Pathological Gambling Measure [41] developed by the authors of the Quinte Longitudinal Study. Further details on this instrument are available in the manual of the QLS [35].

### Statistical analyses

All analyses were carried out using the SPSS for Windows software, version 20. Prevalence rates were described by frequency and percentage of respondents reporting the presence of the given problem behavior. Chi-square tests were used to investigate whether prevalence rates were associated with sex. Cramer’s V was used to express effect size in these analyses. To investigate the severity of behavioral addiction symptoms over time, the repeated measures general linear model (GLM) was used. In each case, analyses were conducted in the subsample of participants who reported the given problem behavior at the first assessment (Time 2 for excessive eating and Time 1 for all other problem behaviors). This way we had the largest sample sizes and the longest follow-up interval possible (descriptive data for behavioral addiction symptoms of respondents with later problem onset are also provided but for clarity reasons, these data were not analyzed statistically to test the effect of time). Partial eta squared was calculated to express effect size in the GLM analyses.

## Results

### Past year prevalence

Table 2 presents sex-stratified prevalence rates for each of the six excessive behaviors. Results of the chi-square tests indicated significant sex differences in the presence of excessive sexual behavior, shopping, and eating at all survey waves, but no sex differences were found in excessive online chatting. A significant association emerged between sex and excessive exercising at Time 1, but this relationship was not significant in the later waves of data collection. Finally, prevalence of problematic video gaming significantly related with sex at the first three assessment points but not in the last two.

**Table 2:** **Past year prevalence data [N (%)] in the five waves of data collection, stratified by sex**

		Wave 1	Wave 2	Wave 3	Wave 4	Wave 5	Average percent
		2006-2007	2007-2008	2008-2009	2010-2011	2011-2012	M (SD)
Exercise	Males	77 (4.1)	30 (1.7)	24 (1.4)	33 (1.9)	26 (1.5)	2.1 (1.1)
	Females	59 (2.6)	31 (1.4)	34 (1.6)	28 (1.3)	29 (1.4)	1.7 (0.5)
	Total	136 (3.3)	61 (1.6)	58 (1.5)	61 (1.6)	55 (1.4)	1.9 (0.8)
	Comorbid substance use^†^	19 (14.0)	11 (18.0)	6 (10.3)	10 (16.4)	9 (16.4)	15.0 (3.0)
Sex	Males	76 (4.1)	45 (2.5)	54 (3.1)	53 (3.1)	46 (2.7)	3.1 (0.6)
	Females	25 (1.1)	20 (0.9)	19 (0.9)	6 (0.3)	13 (0.6)	0.8 (0.3)
	Total	101 (2.5)	65 (1.7)	73 (1.9)	59 (1.5)	59 (1.6)	1.8 (0.4)
	Comorbid substance use^†^	24 (23.8)	17 (26.2)	14 (19.2)	9 (15.3)	13 (22.0)	21.3 (4.2)
Shopping	Males	42 (2.2)	22 (1.2)	28 (1.6)	17 (1.0)	14 (0.8)	1.4 (0.6)
	Females	142 (6.3)	128 (5.9)	127 (5.9)	93 (4.4)	77 (3.7)	5.2 (1.1)
	Total	184 (4.5)	150 (3.8)	155 (4.0)	110 (2.9)	91 (2.4)	3.5 (0.9)
	Comorbid substance use^†^	23 (12.5)	22 (14.7)	16 (10.3)	8 (7.3)	15 (16.5)	12.3 (3.6)
Online chat	Males	34 (1.8)	21 (1.2)	13 (0.7)	20 (1.2)	17 (1.0)	1.2 (0.4)
	Females	28 (1.2)	22 (1.0)	23 (1.1)	14 (0.7)	11 (0.5)	0.9 (0.3)
	Total	62 (1.5)	43 (1.1)	36 (0.9)	34 (0.9)	28 (0.7)	1.0 (0.3)
	Comorbid substance use^†^	13 (21.0)	13 (30.2)	5 (13.9)	7 (20.6)	8 (28.6)	22.9 (6.6)
Video gaming	Males	44 (2.4)	33 (1.9)	33 (1.9)	26 (1.5)	19 (1.1)	1.8 (0.5)
	Females	24 (1.1)	23 (1.1)	23 (1.1)	22 (1.0)	25 (1.2)	1.1 (0.1)
	Total	68 (1.7)	56 (1.4)	56 (1.4)	48 (1.3)	44 (1.2)	1.4 (0.2)
	Comorbid substance use^†^	10 (14.7)	7 (12.5)	7 (12.5)	8 (16.7)	11 (25.0)	16.3 (5.2)
Eating	Males	No data	81 (4.6)	79 (4.5)	76 (4.4)	61 (3.6)	4.3 (0.5)
	Females	No data	210 (9.7)	195 (9.0)	165 (7.8)	140 (6.6)	8.3 (1.4)
	Total	No data	291 (7.4)	274 (7.0)	241 (6.3)	201 (5.3)	6.5 (0.9)
	Comorbid substance use^†^	No data	43 (14.8)	29 (10.6)	23 (9.5)	24 (11.9)	11.7 (2.3)
At least one excessive behavior	Males	230 (12.3)	198 (11.2)	192 (11.0)	192 (11.2)	157 (9.3)	11.0 (1.0)
	Females	228 (10.1)	334 (15.4)	318 (14.7)	262 (12.4)	229 (10.9)	12.7 (2.1)
	Total	458 (11.1)	532 (13.5)	510 (13.1)	454 (11.9)	386 (10.2)	12.0 (1.2)
At least two excessive behaviors	Males	39 (2.1)	30 (1.7)	30 (1.8)	25 (1.5)	20 (1.2)	1.7 (0.3)
	Females	38 (1.7)	80 (3.7)	81 (3.8)	60 (2.9)	49 (2.3)	2.9 (0.9)
	Total	77 (1.8)	110 (2.8)	111 (2.8)	85 (2.3)	69 (1.8)	2.3 (0.5)

^†^Refers to the combined sample of males and females reporting the given excessive behavior in the given data wave.

Although the results above demonstrated that significant differences existed between the female and male subsamples with regard to the occurrence of several problem behaviors, we also displayed prevalence data for the total sample (Table 2) to facilitate comparisons with other studies not using sex stratification. The descriptive data show that the most prevalent problem behavior in both sexes is compulsive overeating (4.3% in men and 8.3% in women), while the least common is excessive online chatting (1.2%) in the male sample and excessive sexual behavior among females (0.8%). It is also worthy of note that the highest prevalence rate for each behavior was observed at the first assessment point (no matter if it was the second wave of the survey as in the case of excessive eating or the first wave as in the case of all other problem behaviors). Table 2 also presents data on the co-occurrence of the six excessive behaviors and the rate of comorbid substance abuse during the study period.

### Persistence and trajectories of symptom severity change

Our data suggested that in the vast majority of cases the reported problem behaviors were transient (Table 3). Within the subsample of respondents reporting a given problem behavior, most participants reported the given excessive behavior only once during the 5-year study period. Even the most stable problem behavior (excessive sexual behavior) was reported five times only by 5.4% of those males who reported having difficulties with this problem behavior. Among females, compulsive shopping proved to be the most stable: but even in this case, 3% of women struggling with this problem behavior reported it five times during the study period.

**Table 3:** **Transiency of the six excessive behaviors studied, stratified by sex**

		Reported any number of times	Reported once	Reported twice	Reported three times	Reported four times	Reported five times
Exercise	Males	159 (100)	133 (83.6)	22 (13.8)	3 (1.9)	1 (1.0)	0 (0.0)
	Females	149 (100)	123 (82.6)	22 (14.8)	2 (1.3)	2 (1.3)	0 (0.0)
	Total	308 (100)	256 (83.1)	44 (14.3)	5 (1.6)	3 (1.0)	0 (0.0)
Sex	Males	167 (100)	106 (63.5)	35 (21.0)	15 (9.0)	2 (1.2)	9 (5.4)
	Females	68 (100)	54 (79.4)	13 (19.1)	1 (1.5)	0 (0.0)	0 (0.0)
	Total	235 (100)	160 (68.1)	48 (20.4)	16 (6.8)	2 (1.0)	9 (3.8)
Shopping	Males	87 (100)	64 (73.6)	13 (14.9)	8 (9.2)	1 (1.1)	1 (1.1)
	Females	338 (100)	209 (61.8)	68 (20.1)	32 (9.5)	19 (5.6)	10 (3.0)
	Total	425 (100)	273 (64.2)	81 (19.1)	40 (9.4)	20 (4.7)	11 (2.6)
Online chat	Males	72 (100)	51 (70.8)	13 (18.1)	4 (5.6)	4 (5.6)	0 (0.0)
	Females	72 (100)	55 (76.4)	12 (16.7)	2 (2.8)	2 (2.8)	1 (1.4)
	Total	144 (100)	106 (73.6)	25 (17.4)	6 (4.2)	6 (4.2)	1 (1.0)
Video gaming	Males	117 (100)	87 (74.4)	25 (21.3)	2 (1.7)	3 (2.6)	0 (0.0)
	Females	84 (100)	66 (78.6)	7 (8.3)	7 (8.3)	4 (4.8)	0 (0.0)
	Total	201 (100)	153 (76.1)	32 (15.9)	9 (4.5)	7 (3.5)	0 (0.0)
Eating	Males	202 (100)	143 (70.8)	32 (15.8)	18 (8.9)	9 (4.5)	N/A
	Females	398 (100)	207 (52.0)	99 (24.9)	63 (15.8)	29 (7.3)	N/A
	Total	600 (100)	350 (58.3)	131 (21.8)	81 (13.5)	38 (6.3)	N/A

Descriptive data for the trajectories of symptom severity change can be seen in Table 4. For these data, we considered each individual who reported an excessive behavior at data waves 1–4. According to the results of the general linear model procedure— conducted in the subsamples of participants only who reported the given problem behavior at the first assessment—, the main effect of time was significant concerning each problem behavior [exercising: F(4) = 92.89, p < 0.001, η^2^ = 0.40; sexual behavior: F(4) = 21.64, p < 0.001, η^2^ = 0.18; shopping: F(4) = 36.96, p < 0.001, η^2^ = 0.17; online chatting: F(4) = 19.63, p < 0.001, η^2^ = 0.25; video gaming: F(4) = 26.94, p < 0.001, η^2^ = 0.29; and excessive eating: F(3) = 21.74, p < 0.001, η^2^ = 0.07], indicating a reliable decreasing trend in symptom severity across time. The magnitude of the main effects was medium or large with regards to all problem behaviors. Results of the post hoc analyses are summarized in Table 5.

**Table 4:** **Descriptive data on symptom severity trajectories for the six problem behaviors [M (SD)]**

		Reported problematic level of involvement at T1	Incidence at T2	Incidence at T3	Incidence at T4
Exercise	T1	2.85 (2.96)	-	-	-
	T2	0.26 (1.35)	3.20 (3.29)	-	-
	T3	0.13 (1.10)	0.57 (2.98)	2.70 (3.11)	-
	T4	0.07 (0.41)	0.46 (1.52)	0.62 (2.10)	2.22 (2.18)
	T5	0.21 (1.03)	0.24 (0.99)	0.07 (0.47)	0.37 (1.52)
	n	136	47	49	40
Sex	T1	3.79 (3.90)	-	-	-
	T2	1.26 (3.26)	4.55 (3.75)	-	-
	T3	0.97 (2.54)	0.51 (1.50)	3.30 (3.27)	-
	T4	1.19 (2.79)	0.20 (1.12)	1.37 (2.99)	2.68 (3.50)
	T5	1.00 (2.68)	0.23 (1.27)	0.59 (2.17)	0.78 (2.14)
	n	101	40	44	26
Shopping	T1	3.97 (3.81)	-	-	-
	T2	1.21 (2.96)	2.80 (3.72)	-	-
	T3	1.25 (3.11)	0.87 (2.43)	2.24 (3.23)	-
	T4	0.75 (2.36)	0.46 (2.02)	0.14 (0.88)	3.30 (4.40)
	T5	0.71 (2.42)	0.61 (2.11)	0.41 (1.65)	0.16 (1.04)
	n	184	99	71	45
Online chat	T1	3.48 (3.52)	-	-	-
	T2	1.25 (3.24)	4.17 (4.00)	-	-
	T3	0.42 (1.78)	1.01 (2.38)	3.64 (3.56)	-
	T4	0.80 (2.40)	0.59 (2.22)	0.40 (1.51)	3.59 (3.27)
	T5	0.73 (2.20)	0.24 (0.99)	0.05 (0.23)	0.72 (2.68)
	n	62	30	23	17
Video gaming	T1	4.13 (3.63)	-	-	-
	T2	1.51 (3.20)	4.01 (3.39)	-	-
	T3	0.56 (2.07)	1.02 (2.51)	4.40 (3.82)	-
	T4	0.53 (2.31)	1.25 (3.82)	1.01 (3.38)	3.26 (3.34)
	T5	0.50 (1.96)	1.07 (2.84)	0.00 (0.00)	0.51 (1.76)
	n	68	40	39	31
Eating	T2	-	3.29 (3.68)	-	-
	T3	-	1.85 (3.43)	2.94 (3.41)	-
	T4	-	1.33 (2.92)	1.06 (2.53)	2.96 (3.48)
	T5	-	1.39 (2.92)	0.94 (2.57)	0.80 (2.47)
	n	-	291	153	98

**Table 5:** **Results of the post hoc tests for the main effect of time on symptom severity**

Exercise	I > II (2.67), I > III (2.82), I > IV (2.90), I > V (2.75), II ≈ III (0.14), II ≈ IV (0.22), II = V (0.08), III = IV (0.08), III = V (−0.07), IV = V (−0.15)
Sex	I > II (2.37), I > III (2.74), I > IV (2.59), I > V (2.72), II = III (0.37), II = IV (0.22), II = V (0.35), III = IV (−0.15), III = V (−0.02), IV = V (0.13)
Shopping	I > II (2.75), I > III (2.70), I > IV (3.04), I > V (3.16), II = III (−0.06), II = IV (0.28), II = V (0.41), III = IV (0.35), III = V (0.46), IV = V (0.12)
Online chat	I > II (2.30), I > III (3.07), I > IV (2.69), I > V (2.79), II > III (0.77), II = IV (0.40), II = V (0.49), III = IV (−0.38), III = V (−0.28), IV = V (0.10)
Video gaming	I > II (2.60), I > III (3.44), I > IV (3.48), I > V (3.63), II > III (0.84), II ≈ IV (0.88), II > V (1.04), III = IV (0.04), III = V (0.20), IV = V (0.16)
Eating	II > III (1.33), II > IV (1.68), II > V (1.82), III = IV (0.34), III > V (0.49), IV = V (0.15)

Note. Roman numbers stand for data from survey waves 1 to 5, respectively. Equal sign means p ≥ 0.1, almost equal sign means 0.05 ≥ p < 0.1. Numbers in parentheses are mean differences.

**Figure 1:**
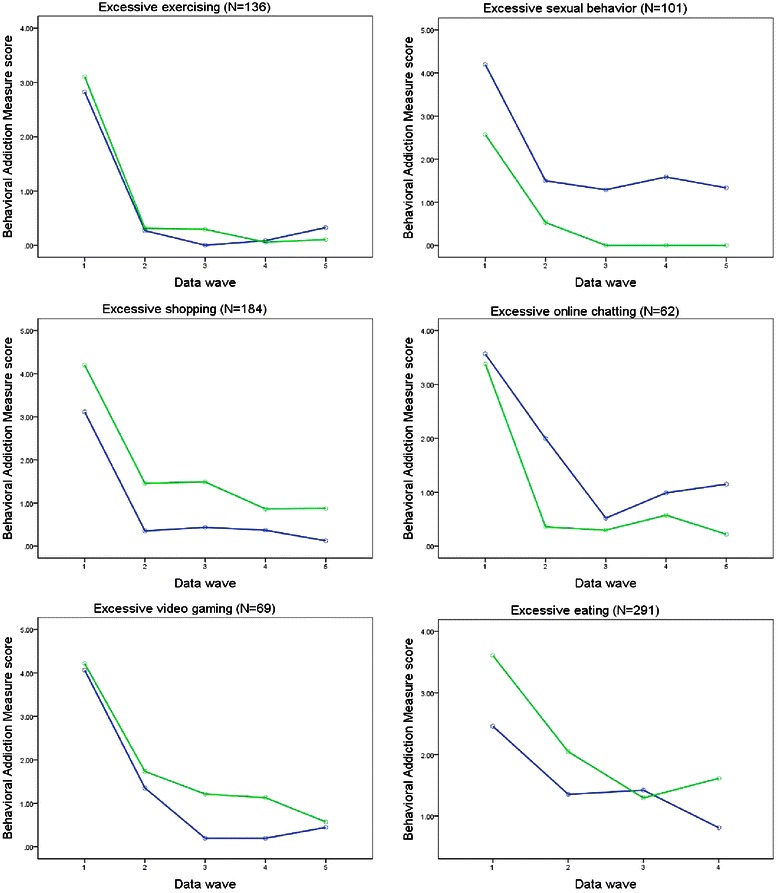
**Mean trajectories for the symptom scores of the six excessive behaviors studied, stratified by sex.** Note. Data of males are displayed in blue/dark, those of females in green/bright.

Figure 1 shows a sex-stratified graphical representation of the mean trajectories for the Behavioral Addiction Measure scores for all problem behaviors studied in individuals who reported an excessive behavior at data wave 1 (or data wave 2 in the case of excessive eating). The main effect of sex concerning the mean trajectories was significant concerning excessive sexual [F(1) = 6.51, p = 0.012, η^2^ = 0.06], shopping [F(1) = 6.76, p = 0.010, η^2^ = 0.04], and eating [F(1) = 4.70, p = 0.031, η^2^ = 0.02] behavior but not in the case of excessive exercising [F(1) = 0.21, p = 0.647, η^2^ < 0.01], online chatting [F(1) = 2.09, p = 0.153, η^2^ = 0.03], and video gaming [F(1) = 1.44, p = 0.234, η^2^ = 0.02]. The effect sizes of even the significant sex main effects were small, indicating that symptom severity for males and females were not substantially different.

The time × sex interaction was insignificant in each model [exercising: F(4) = 0.71, p = 0.482, η^2^ < 0.01; sexual behavior: F(4) = 0.28, p = 0.824, η^2^ < 0.01; shopping: F(4) = 0.38, p = 0.795, η^2^ < 0.01; online chatting: F(4) = 1.19, p = 0.314, η^2^ = 0.02; and video gaming: F(4) = 0.53, p = 697, η^2^ < 0.01] – with the exception of excessive eating where a weak tendency emerged [F(3) = 2.29, p = 0.077, η^2^ = 0.01]. The non-significant interaction terms indicate that the basic pattern of decreasing symptom severity is not different for males and females.

### Help seeking

Results on help seeking are not presented in sex stratification as frequencies were too low to conduct comparisons. The data show that help seeking is infrequent in the case of excessive sexual behavior, shopping, net chatting, and video gaming (Table 6). However, help seeking was substantially more prevalent in the case of excessive eating and exercising but even concerning these problem behaviors, the rate of those actively looking for help was less than half of those reporting the problem. A further important aspect of the results is that the rates for seeking any versus professional help were not substantially divergent, indicating that the majority who sought help turned to health care professionals and not only to informal resources as family or friends.

**Table 6:** **Rate [N (%)] of help seeking in the five waves of data collection**

	Wave 1		Wave 2		Wave 3		Wave 4		Wave 5		Average percent M (SD)	
	Any kind	Professional	Any kind	Professional	Any kind	Professional	Any kind	Professional	Any kind	Professional	Any kind	Professional
Exercise	8 (25.0)	7 (21.9)	5 (26.3)	4 (21.1)	5 (38.5)	5 (38.5)	8 (72.7)	8 (72.7)	6 (46.2)	4 (30.8)	45.6 (20.1)	37.0 (21.2)
Sex	5 (13.2)	5 (13.2)	2 (8.0)	1 (4.0)	4 (14.3)	2 (7.1)	2 (8.0)	2 (8.0)	3 (13.0)	2 (8.7)	11.4 (2.9)	8.2 (3.3)
Shopping	6 (7.6)	4 (5.1)	9 (17.3)	4 (7.7)	3 (5.8)	3 (5.8)	6 (20.0)	4 (13.3)	2 (7.7)	2 (7.7)	10.3 (6.5)	7.9 (3.2)
Online chat	1 (4.8)	1 (4.8)	5 (25.0)	3 (15.0)	2 (15.4)	2 (15.4)	1 (7.1)	1 (7.1)	2 (18.2)	2 (18.2)	16 (10.3)	12.1 (5.8)
Video gaming	2 (8.3)	1 (4.2)	4 (16.0)	2 (8.0)	1 (4.3)	1 (4.3)	1 (5.9)	1 (5.9)	1 (7.1)	0 (0.0)	9.5 (5.9)	4.5 (2.9)
Eating	No data	No data	30 (33.7)	25 (28.1)	34 (37.0)	28 (30.4)	31 (38.3)	27 (33.3)	34 (47.2)	24 (33.3)	40.5 (9.5)	31.3 (2.5)

Note: Professional help included assistance from a family physician, psychologist, psychiatrist, counseling service, or telephone help/hotline.

## Discussion

As reflected in the definition of addiction provided by the American Society of Addiction Medicine [44], the concept of addiction has expanded in recent years to include many types of excessive behaviors, not only those related to the misuse of substances. The aim of the present study was to contribute to a better understanding and classification of some of these behaviors by describing their natural course. We also wanted to explore whether sex differences—often described concerning many aspects of substance addictions [45,46]—could be observed in this regard.

Our results showed that the prevalence of the excessive behaviors studied was highly variable across the individual disorders, with the highest value reported for excessive eating for both women and men. Despite some oversampling for individuals at risk for a behavioral addiction (gambling disorder), the prevalence rates found in the present study – with the exception of excessive eating [47] – tended to be lower than those reported in most other Canadian studies [34,36,48]. However, this difference is due to the focus on students in two of these studies [34,48] and the use of an online panel in the other study [36], both of which are known to have higher rates of excessive behaviors [49,50]. The one study that is directly comparable is a 2010 landline telephone survey of 2,000 Albertans [36], which also used a singular question to establish past year prevalence of various behavioral addictions. The obtained rates of excessive shopping, sex, and video game addiction were very similar in these two surveys: shopping (5.2% in Alberta versus 4.5% in QLS); sex (3.0% in Alberta versus 2.5% in QLS); and video game addiction (2.6% in Alberta versus 1.7% in QLS). When comparing the present prevalence rates with international data [1,9,51-57], the general trend is that respondents of the QLS reported excessive behaviors less frequently than participants of other surveys, although again, the large variability in sample characteristics and assessment methodology makes direct comparison difficult.

Similar to substance addictions, significant sex differences were found in the prevalence rates of several excessive behaviors. In line with previous data [9,34,47,52,54], excessive eating was almost twice and excessive shopping more than three times more prevalent among females than males, while the prevalence of excessive sexual behavior was almost four times higher in males. It is worth mentioning, however, that other European investigations have not revealed significant differences between males and females in the prevalence rate [53] or motives [58] for excessive buying. In addition, the sex differences in the prevalence of excessive exercising and problematic video gaming were not observed consistently in the present study, while the association of the respondents’ sex with the occurrence of online chatting was insignificant in each survey wave. This patterning of the data calls our attention to the importance of considering sex and gender differences when collecting information on behavioral addictions or planning prevention and intervention strategies to help cope with them.

The results concerning the trajectories of symptom severity over time showed that the excessive behaviors in this population were not steady: they were episodic rather than continuous in nature. Contrary to prevalence data, this phenomenon was independent of the respondents’ sex in the case of each problem behavior. The present findings on the transient nature of excessive behaviors are in line with the very few previous studies showing that problem gambling, excessive eating, and video gaming are less persistent [31,35,59-64] than traditional substance-related addictions [65,66]. The only findings in the literature that systematically contradict with the present results are related to excessive shopping, which was stable in about half of the individuals. However, the studies reporting these data are typically more than two decades old, are based on almost exclusively female samples with significant psychiatric comorbidities, and utilized retrospective or short-term prospective designs [67-69].

Similar to the results of previous studies on help-seeking with regards to gambling disorders [70], the findings of the present investigation showed that individuals struggling with excessive behaviors sought professional help relatively rarely. Exceptions were excessive exercising and eating concerning which about one third of those reporting the problem also stated that they sought professional help to cope with their problem. However, no systematic association was found between decrease in symptom severity and professional help-seeking: while the main effect of time was the strongest for excessive exercising, in the same time it was the weakest for excessive eating. Also, while 83.1% of those reporting excessive exercising reported this problem only once (meaning a recovery within a year), this could be said of only about 58.3% of excessive eaters. Another interesting aspect of the findings on help-seeking is that most people who sought help for their excessive behaviors turned to health care professionals and not to lay helpers or social support in general. This might indicate that individuals looking for help with excessive behaviors rather perceive their problem as a medical one and not as a lifestyle, spiritual or other issue (although for example psychologists can be approached with lifestyle issues as well).

The overall patterning of the results concerning help-seeking and symptom trajectories over time suggests that the largest part of the change in symptom severity of excessive behaviors in this study is to be considered as spontaneous and not (professionally) assisted recovery. This aspect of our results is against a conceptualization of addictions as progressive without treatment (e.g., American Society of Addiction Medicine [44]). Further prospective research is needed to demonstrate the spontaneous nature of recovery from excessive behaviors more clearly (in the present study, questions referred to life-time and not recent/current help-seeking—not providing the opportunity to investigate the specific role of help-seeking in recovery). Also, future research is needed to investigate whether the spontaneous recovery from problem behaviors is typically permanent or whether affected individuals “switch” to other types of dysfunctional behaviors, which is a reasonable possibility cf. [71] not investigated in the present study.

Strengths of the present research include its prospective design, thereby reducing problems emerging from recall; the community sampling approach, which decreases selection biases inherent in pure clinical samples; the 5-year follow-up period, which provided sufficient time for longer-term changes to be observed; and the very high retention rate. However, some limitations of the present study should also be noted. First, although the total sample was quite large, sample sizes for the analyses concerning trajectories of symptom severity over time were relatively small. Further, the present sample rather should be seen as a general population sample (with a mean age of 46.1 years) limiting the generalizability of the present findings concerning spontaneous remission in age groups more at-risk for developing addictions (e.g., adolescents) or in clinical populations, where more severe symptomatology and higher number of comorbid disorders are characteristic. Also, the more frequent occurrence of excessive exercising and video gaming among those not followed through all five data waves reduces the validity of the data concerning these behaviors. In addition, although previous research indicated that self-identification through a single question for an excessive behavior is a reliable and clinically meaningful tool in indentifying persons with addictive disorders, these studies concentrated on substance addictions, pathological gambling, and video gaming. The generalizability of the methodological appropriateness of this assessment method is, therefore, questionable. Further, a detailed psychometric investigation of the Behavioral Addiction Measure for use with behaviors other than gambling also remains to be done.

Finally, the phenomenon that the prevalence rate of each excessive behavior was the highest at its first assessment requires some explanation. One possibility is that respondents over-reported at the first assessment, and/or started to underreport their problems in the later survey waves (either because they felt less anonymous or to avoid the large number of extra questions resulting from reporting each additional excessive behavior). However, a reporting bias is contraindicated to some extent by the fact that lifetime reports of gambling addiction were unchanged from assessment 1 to assessment 2 (data not presented in this paper). Another possibility is that because the sample was a non-clinical one, the survey itself may have served as a first intervention for many of the respondents. The repeated investigation of excessive behaviors—by raising participants’ awareness—could lead to true changes in behavior; thus indicating that the consistent decreasing trend in problem severity is a reliable result of the present study and not a consequence of measurement bias. A final possible explanation is that the QLS over-selected for an inherently unstable entity (i.e., problem gambling), resulting in the highest problem gambling prevalence rate in the first assessment and the highest remission occurring between assessment 1 and 2. Because behavioral addictions tend to co-occur [1], this selection procedure likely also resulted in over-selection of the similarly unstable behavioral addictions that were the focus of the present investigation. This would then produce the same result: artificially high prevalence in the first assessment with accentuated remission in the later assessments.

## Conclusions

The present study provides preliminary information on the occurrence and episodic nature of excessive exercising, sexual behavior, shopping, online chatting, video gaming, and eating in the Ontario population. These data can aid the better understanding and classification of and intervention planning for excessive behaviors.

## Additional file

Additional file 1: **Full text of the Behavioral Addiction Measure.** The file contains the full text of an instrument used in the present study.
