# Supplementary material for: Natural course of behavioral addictions: a 5-year longitudinal study
Source: BMC Psychiatry. 2015 Jan 22;15:4. doi: 10.1186/s12888-015-0383-3 (PMC4320621; doi:10.1186/s12888-015-0383-3)
Supplement: Additional file 1: — Full text of the Behavioral Addiction Measure. The file contains the full text of an instrument used in the present study. [file 12888_2015_383_MOESM1_ESM.doc]

1. How often have you engaged in [problem behavior] in the past 12 months?

- several times a day (1)
- daily (1)
- several times a week (1)
- a few times a week (1)
- once a week (0)
- a few times a month (0)
- once a month or less (0)

2. Has your involvement in this activity caused significant financial concerns for you or someone close to you in the past 12 months?

- yes (1)
- no (0)

3. Has your involvement in this activity either caused you to borrow a significant amount of money or sell some of your possessions in the past 12 months?

- yes (1)
- no (0)

4. Has your involvement in this activity caused significant mental stress such as anxiety, depression, or paranoia for you or someone close to you in the past 12 months?

- yes (1)
- no (0)

5. Has your involvement in this activity caused serious problems in your relationship with your spouse/partner, or important friends or family in the past 12 months?

- yes (1)
- no (0)

6. Has your involvement in this activity caused you to repeatedly neglect your children or family in the past 12 months?

- yes (1)
- no (0)

7. Has your involvement in this activity caused you or someone else to have significant health problems or to be injured in the past 12 months?

- yes (1)
- no (0)

8. In the past 12 months, have you repeatedly engaged in this activity in situations or ways that are physically dangerous?

- yes (1)
- no (0)

9. Has your involvement in this activity caused significant work or school problems for you or someone else in the past 12 months?

- yes (1)
- no (0)

10. Has your involvement in this activity caused you to miss a significant amount of time off work or school in the past 12 months?

- yes (1)
- no (0)

11. Has your involvement in this activity caused you or someone close to you to commit illegal acts in the past 12 months?

- yes (1)
- no (0)

12. Has your involvement in this activity caused you or someone close to you to have legal problems in the past 12 months?

- yes (1)
- no (0)

13. Is there anyone else who would say that your involvement in this activity has caused any significant problems for you or someone close to you in the past 12 months, regardless of whether you thought it was true?

- yes (1)
- no (0)

14. In the past 12 months, have you engaged in this activity for a longer time, or more frequently than you intended to?

- yes (1)
- no (0)

15. In the past 12 months, have you made attempts to either cut down, control or stop your involvement in this activity?

- yes (1)
- no (0)

16. Were you successful in these attempts?

- yes (1)
- no (0)

17. In the past 12 months, did you experience irritability, restlessness, strong cravings or other withdrawal symptoms when you reduced or stopped engaging in this activity?

- yes (1)
- no (0)

18. In the past 12 months, did you have strong cravings for the activity?

- yes (1)
- no (0)

19. In the past 12 months, is there anyone else who would say that you either had strong cravings for this activity or experienced a loss of control over your behaviour?

- yes (1)
- no (0)

20. In the past 12 months, did you spend a great deal of time thinking about or doing things related to this activity?

- yes (1)
- no (0)

21. In the past 12 months, did you find that you had to engage in this activity more and more to get the same effect you wanted?

- yes (1)
- no (0)
